# A Web-Based Peer Support Network to Help Care Partners of People With Serious Illness: Co-Design Study

**DOI:** 10.2196/53194

**Published:** 2024-05-08

**Authors:** Elizabeth A O’Donnell, Aricca D Van Citters, Inas S Khayal, Matthew M Wilson, David Gustafson, Amber E Barnato, Andrea C Buccellato, Colleen Young, Megan M Holthoff, Eugene Korsunskiy, Stephanie C Tomlin, Amelia M Cullinan, Alexandra C Steinbaugh, Jennifer J Hinson, Kristen R Johnson, Andrew Williams, Ruth M Thomson, Janet M Haines, Anne B Holmes, Ann D Bradley, Eugene C Nelson, Kathryn B Kirkland

**Affiliations:** 1 Communications, Marketing and Community Health Alice Peck Day Memorial Hospital Lebanon, NH United States; 2 The Dartmouth Institute for Health Policy & Clinical Practice Geisel School of Medicine at Dartmouth Lebanon, NH United States; 3 Dartmouth Cancer Center Geisel School of Medicine Dartmouth Lebanon, NH United States; 4 Palliative Medicine Geisel School of Medicine at Dartmouth Lebanon, NH United States; 5 Section of Palliative Medicine Dartmouth Health Lebanon, NH United States; 6 College of Engineering University of Wisconsin Madison, WI United States; 7 Mayo Clinic Connect Mayo Clinic Rochester, MN United States; 8 Thayer School of Engineering Dartmouth College Hanover, NH United States; 9 Patient and Family Advisors Dartmouth Health Lebanon, NH United States

**Keywords:** human-centered design, caregivers, care partners, serious illness, peer support, online support network, virtual network, online network, caregiver, unmet need, unmet needs, active care, bereaved care, bereavement, clinician, clinicians, function, functions, specification, information, emotional support, technical support, privacy protection, rural, viability, impact, engineering design, care provider, care providers, mortality, quality of life, tertiary care, caregiving

## Abstract

**Background:**

Care partners of people with serious illness experience significant challenges and unmet needs during the patient’s treatment period and after their death. Learning from others with shared experiences can be valuable, but opportunities are not consistently available.

**Objective:**

This study aims to design and prototype a regional, facilitated, and web-based peer support network to help active and bereaved care partners of persons with serious illness be better prepared to cope with the surprises that arise during serious illness and in bereavement.

**Methods:**

An 18-member co-design team included active care partners and those in bereavement, people who had experienced serious illness, regional health care and support partners, and clinicians. It was guided by facilitators and peer network subject-matter experts. We conducted design exercises to identify the functions and specifications of a peer support network. Co-design members independently prioritized network specifications, which were incorporated into an early iteration of the web-based network.

**Results:**

The team prioritized two functions: (1) connecting care partners to information and (2) facilitating emotional support. The design process generated 24 potential network specifications to support these functions. The highest priorities included providing a supportive and respectful community; connecting people to trusted resources; reducing barriers to asking for help; and providing frequently asked questions and responses. The network platform had to be simple and intuitive, provide technical support for users, protect member privacy, provide publicly available information and a private discussion forum, and be easily accessible. It was feasible to enroll members in the ConnectShareCare web-based network over a 3-month period.

**Conclusions:**

A co-design process supported the identification of critical features of a peer support network for care partners of people with serious illnesses in a rural setting, as well as initial testing and use. Further testing is underway to assess the long-term viability and impact of the network.

## Introduction

Care partners of people with serious illnesses are often overlooked and poorly understood by health care professionals, lack support and educational resources, and are likely to experience significant challenges and unmet needs [1,2]. For many, the work of caring for a person with a serious illness can bring deep satisfaction and can also be challenging [3]. Care partners experience burdens in every area of their lives—emotional, physical, social, spiritual, and financial [2,4,5]. The death of someone with a serious illness (as well as the events leading up to it) also brings hardship, including stress related to loneliness, grief, trauma, role recognition, and self-identity [6-9]. Social isolation and grief are strongly correlated with subsequent depression and related symptoms in bereaved spouses, including sadness, appetite loss, and lower quality of life [5,10]. Bereaved care partners may also face challenges navigating household, financial, social planning, and legal affairs, as well as reintegrating into their local community [11] and accessing available resources. Addressing care partner needs has become a pressing health, economic, and social imperative [12].

Increasingly, care partners are joining online peer support networks to obtain emotional support, access information, and connect and share with others in similar circumstances [13-17]. While people often prefer connecting with health professionals for medical information, they prefer connections with peers over professionals for accessing emotional support or practical advice [18]. In the case of serious illness, when patients may not be well enough to use online peer support networks themselves, care partners are more likely to participate [19].

Previous research has demonstrated a number of variables that contribute to an online peer support network’s success or failure [20-22]. Communities that have a clear, defined purpose; foster a strong sense of community; and have a high level of activity are more likely to be successful [20]. Additionally, sustained organizational and financial support for maintaining an online community from inception to maturity is essential, including support for a community manager who sets the tone for the community, creates content, conducts outreach, and fosters a sense of community [20,23]. Successful online networks also harness the interests and abilities of their users to strengthen the community. Networks with more active users are generally more successful [21] because they maintain a critical mass to allow for diversity in experiences and individual attributes, allowing for the natural formation of relationships and answering questions.

Evidence on the impact of online peer support networks for care partners is promising [14,24-26]. For care partners of people with cancer, studies show evidence of decreased care partner emotional distress [27], negative mood [28,29], and sense of burden [29], as well as increases in quality of life and self-efficacy [27]. For care partners of people with dementia, online networks can lead to improvement in self-efficacy [30], decision-making confidence [31], and care partner and patient relationship quality [32]. Care partners also benefit from being able to freely express their sentiments and provide mutual support in a dedicated digital space apart from their loved ones [33,34]. Even people who observe network activity without participating report that reading about the experiences of others is empowering and informative [35,36]. Online networks offer certain advantages: 24/7 home access, flexibility (communication is often asynchronous), anonymity, and a wide range of expertise and experience not limited by geography [37-39].

Online peer support networks for care partners often target specific health conditions (eg, breast cancer and Alzheimer dementia) or stages of caregiving (treatment vs bereavement), but infrequently support care partners of people with diverse conditions or the transition between stages of caregiving. They may also fail to provide active facilitation and moderation; identify and vet regional resources and support from local peers; or provide the possibility of meeting in person. A co-design process can elicit those factors that matter most to the people for whom the network is intended to serve and ensure the successful adoption of the proposed solution [40].

The objective of this paper is to describe a co-design process and the resulting key functions and specifications for a regional, facilitated, and web-based peer support network that can meet the needs of active and bereaved care partners of persons with serious illnesses.

## Methods

### Overview

**Figure 1:**
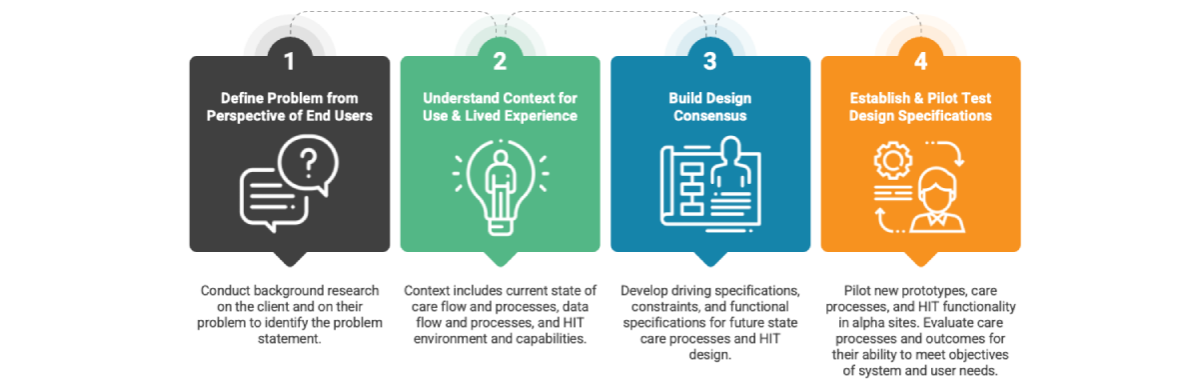
Co-design framework (reproduced from The Dartmouth Institute for Health Policy & Clinical Practice, which is published under Creative Commons Attribution 4.0 International License). HIT: health information technology.

We applied a co-design framework (Figure 1 [41,42]), which combines human-centered design [43] and engineering design [44,45] processes, to create the specifications for a regional, facilitated, and web-based peer support network. The framework includes 4 stages: defining the problem, understanding the context for use, developing and building consensus around functions and specifications that fulfill identified needs, and establishing and pilot-testing design specifications.

### Ethical Considerations

The study was approved by the Dartmouth College institutional review board (#2000907).

A waiver of written informed consent was used for the surveys, as the only link to the survey respondent would have been the written informed consent document. No identifiable information was collected and individuals were not paid for participating in human subjects research.

### Target Population

The target population, hereafter referred to as end users, was defined as care partners (ie, informal caregivers or family members) supporting or providing care to adults (aged 18 years or older) with a serious illness and those who have experienced the loss of someone to a serious illness. The term care partner was chosen by the co-design team to reflect their role and relationship in partnering with a person with a serious illness. Serious illness has been defined as one that carries a high risk of mortality and either negatively impacts a person’s daily function or quality of life, or excessively strains their care partners [46]. Care partners include relatives, spouses or partners, friends, neighbors, or others who have a significant personal relationship with, and who provide a broad range of assistance to, a person with a serious illness. We focused on care partners living in New Hampshire and Vermont, the catchment area for Dartmouth-Hitchcock Medical Center.

### Participants

We formed an 18-member team to co-design the network. The team included 2 active and bereaved care partners of people with serious illness, 4 adults with serious illness, 6 interdisciplinary palliative care clinical team members, and 6 support service staff*.* Care partners and people with serious illnesses were recruited by our clinical team partners. Clinical team members were affiliated with Dartmouth-Hitchcock Medical Center, a rural tertiary care academic medical center in New Hampshire. Facilitation of the co-design process was led by researchers with expertise in co-design, evaluation, and quality improvement (EAO and ADVC). The design process was informed by consultation with an expert in human-centered design (EK) and a systems engineer (ISK). To ensure our design aligned with best practices, we consulted with external advisors with expertise in facilitated support networks (DG and CY), met with regional health care and support partners, and obtained input from an external advisory committee with expertise in scaling innovations, business, and serious illness.

The co-design team met twice a month for 8 months (April to November 2019) to identify and prioritize the functions and specifications of the network and met monthly for 6 months (December 2019 to May 2020) to test prototypes.

### Defining the Problem and Understanding Context for Use

**Figure 2:**
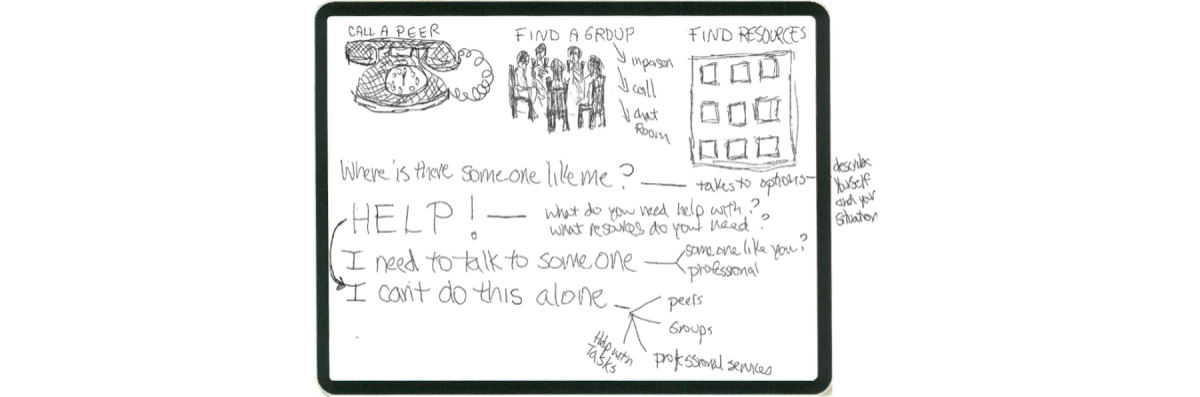
Sketch of a serious illness community network, created by a co-design team member.

We conducted human-centered design exercises [43] to elicit community needs and assets, define the problem, and understand the context for use. We drew upon stories of serious illnesses shared by care partners to identify needs arising from lived experiences. Design facilitators shadowed [43] outpatient palliative care visits and attended interdisciplinary palliative care team meetings to further understand the context of use; services provided by the care team; and the daily lives of people with serious illness, their care partners, and clinicians [47]. We developed empathy maps [43] to reflect and articulate what end users hear, see, say, do, think, and feel, and to identify points of pain and gain. We used a visual thinking exercise [48] to sketch ideas for an ideal support network (example, Figure 2). Design exercises were reviewed during design sessions to discuss critical functions and important features that fulfill and support these functions.

We supplemented design activities with surveys of potential end users. Between December 2018 and April 2019, we collected 28 surveys from a convenience sample of active care partners presenting at the Dartmouth-Hitchcock Medical Center outpatient palliative care clinic and 21 surveys from a convenience sample of bereaved care partners affiliated with the clinic or regional health organizations (eg, hospice) to elicit information on the challenges, needs, and desire for peer connection among active and bereaved care partners (surveys are provided in Multimedia Appendix 1). We used descriptive statistics to summarize categorical data, and thematic analyses to identify themes from open-ended questions.

### Building Design Consensus

We collated, organized, and systematically described the identified needs into a hierarchical list. We converted the highest-level needs into functions for the network to achieve. At the highest level, for example, “care partner need for information” was converted to “connecting care partners to information” and “care partner need for emotional support” was converted to “facilitating emotional support.” We identified potential specifications associated with each function of the network.

Co-design team members independently rated the importance of each specification using a five-item prioritization scale: (5) must have, (4) high importance (feasible without), (3) should have (very important), (2) could have (consideration), and (1) desirable (will not have at this time) [49]. Team members were provided an opportunity to describe their understanding and thought process for preference identification as a group. Scores were weighted and averaged by respondent type to ensure that patients and care partners, clinicians, and support service staff had equal participation weight (eg, scores from 6 clinicians were averaged to create 1 clinician average).

### Pilot-Testing Design Specifications

We tested a series of prototypes to identify the importance of different network components. These included storytelling exercises, face-to-face group conversations, one-to-one matching of care partners, group videoconferencing, an online discussion forum, and a “Caregiver Day” event at the health center. Summaries are provided in Multimedia Appendix 2.

After finalizing design functions and specifications, we identified potential vendors to host the network by conducting an environmental scan of web-based networks focused on people with serious illnesses and care partners and mapping desired design specifications to vendor capabilities. Four vendors provided demonstrations of their platforms between April and May 2020. Vendor selection was driven by the vendor’s ability to provide the prioritized functions and specifications, the cost to build and maintain the network, and being a US-based company. Following vendor selection, the web-based network platform was customized by the design team to deliver upon design functions and specifications.

User acceptance testing was conducted between April and July 2021. Four care partners and 2 people who previously had a serious illness were invited to register as founding members of the network in April. These founding members were encouraged to invite care partners they knew into the web-based community to test the feasibility of enrolling members. Three clinical champions (physicians and social workers), a chaplain, and a staff member who manages complementary care programs referred care partners to the network. The research team met weekly with the vendor during the user acceptance testing period to resolve issues. The web-based platform remained available for registered members to use while issues were addressed. The network moderator met monthly with the design team to plan future improvements using a quality improvement framework [50].

## Results

### Problem Definition

The co-design process led to clarity around the objective of the network: to help care partners cope with the surprises that arise during serious illness and bereavement. The network, named “ConnectShareCare,” was intended to supplement existing services, to be provided outside of clinical encounters with the health care system or regional professional support and service organizations, and to tap into the wisdom of those with lived experiences.

If successful, the design team anticipated that the network would benefit 4 groups, as outlined in Table 1.

**Table 1:** Anticipated impact of ConnectShareCare.

Audience	Anticipated objectives
Care partners	Improve access to information that can guide decision-making Improve sense of empowerment in making decisions and providing support Decrease sense of distress and social isolation
Community partners	Improve understanding of needs and gaps in service Provide a system to share assets or resources
Clinicians or health care system	Address gaps in services that are not currently met Improve availability to see patients who seek services Improve efficiency of health care encounters
Quality improvement leaders and researchers	Improve understanding of the needs of care partners Align services with care partners and community needs Demonstrate a positive impact of the network over time

### Context for Use and Lived Experiences

Our co-design process identified that active and bereaved care partners have different needs but have common interests in sharing information and providing or receiving support. Active care partners who completed an assessment survey were most challenged by emotional difficulties (eg, worry, uncertainty, or lack of control; 12/28, 43%), providing care and emotional support (7/28, 25%), and practical matters (6/28, 21%). Bereaved care partners were most challenged by loneliness (10/21, 48%), managing grief and emotional difficulties (6/21, 29%), and managing practical matters (5/21, 24%). Active care partners were most helped by support from friends, family, or other social connections (12/28, 43%), as well as by medical professionals (9/28, 32%), while bereaved care partners were most helped by support from friends, family, or other social connections (16/21, 76%) and by developing self-care strategies that led to personal resilience and growth (12/21, 57%).

Most active (18/27, 67%) and bereaved (18/21, 86%) care partners were interested in 1 or more forms of connecting with other people who have shared a similar care experience. Both active and bereaved care partners anticipated that a network could provide support, knowledge, and resources but anticipated challenges associated with time to participate and with forming personal connections.

The series of human-centered design exercises and interviews led to additional insights. First, active and bereaved care partners may benefit from connecting and sharing information with each other. Peer-generated information from care partners who have shared a similar experience feels more authentic, detailed, and actionable. Second, care partners wished to belong to a local support community that was connected through geographic proximity and could provide recommendations for local resources. Third, a web-based network enables care partners to access information at any place or time, allows anonymity, improves access for people who are home-bound or grieving, and may reach an increased number of care partners. Fourth, trained staff who can moderate, promote, and manage the web-based community and volunteers who can recruit and engage users are important. Paid or volunteer moderators can play an important role in listening, making connections, and highlighting information, services, and programs. Fifth, a web-based network would benefit from supplemental opportunities for the community to meet face-to-face or through digital programming.

The design process also identified several potential risks and possible mitigation strategies. First, there was a risk of causing harm to vulnerable end users if the design failed to provide a safe and supportive environment, protect the privacy of sensitive information, or enact acceptable data ownership guidelines. Second, there was a risk associated with the usability of the network among end users who were less facile with web-based services. Third, there was a risk associated with the inability to form a personal connection with peers through a web-based network. Other potential risks included those associated with competition from other networks, inaccurate content or information, and care partners having minimal time to participate in a web-based support network due to other responsibilities.

### Build Design Consensus

Two primary functions emerged from the design activities: to support care partners in (1) providing each other with emotional support and (2) exchanging helpful information and resources. We developed specifications related to these functions (Table 2), as well as the form of the network, including the user interface, data and security, and other considerations (Table 3). 

**Table 2:** Prioritization ranking of design specifications associated with network functions (weighted average scores across patients and families [n=5]; clinicians [n=4]; clinical support service providers [n=5]; and network advisors [n=2]).

Network functions and prioritization ranking of associated design specifications				Ranking^a^
**Function 1: provide each other with emotional support**				
	**Must have**			
		Provide a supportive and respectful space Ability for established “Guidelines and Ground Rules” to be clearly visible to users Ability to protect individual identity (opt-out options for sharing personal information; opportunity to keep geographic location private) Allows moderator functionality for policing interactions and blocking users if necessary	5.00	
	**Should have (very important)**			
		Incorporate and help facilitate, one-to-one connections Ability to locate “true peer” (similar users) through the platform via matching on similar life circumstances (through backend algorithm or user profile details: type of loss, disease, time caregiving, or time since loss) Allow for private one-to-one messaging to facilitate a more personal connection, not monitored by an external entity	3.27	
		Includes opportunity for storytelling based on personal user content or experience	3.00	
		Provides an opportunity to share solutions	3.00	
	**Could have (consideration)**			
		Differentiate between whether people want to feel heard or want to hear solutions Ability for users to designate whether they are looking to hear solutions or feedback or simply share	2.88	
**Function 2: exchange helpful information and resources**				
	**Must have**			
		Provide connections to trusted and curated local, national, and international resources Ability to host webinars in order to share educational content Ability for newsfeed or wall that features newly published content Provide document or resource repository related to user needs Ability to highlight and share local events via calendar, bulletin board, or other Robust search function available to find targeted resources within the platform	4.56	
	**High importance (feasible without)**			
		Reduce the difficulty of asking for help by normalizing needing help Ability to add a button in various locations that asks “Having a hard time asking for help?” and that opens a new page that contains tips or guidelines on how to ask for help and what to expect when asking for help	3.88	
		Provide frequently asked questions list and answers Site provides a list, or ability to create a list, of the most popular or frequently asked questions and answers (eg, “How do I cope with stress?”)	3.76	
	**Should have (very important)**			
		Ability to identify most common needs Ability to organize conversations around themes (or topics) and make it easy for someone with a specific question, topic, or theme to locate information pertaining to it Ability to “like,” (showing interest, support) posts, topics, or comments so that users can see which posts are popular and most useful Ability to follow a discussion thread, topic, etc. Once a user has “followed” something or someone, they can receive a notification when there is new content posted Ability to bookmark posts (to save content) that users would like to revisit	3.47	
		Ability to be supported by local or regional expert moderator (community manager)	3.38	
		Include the ability to publish videos related to the content of the network Allow users to record and post videos instantly (personal and other)	3.06	

^a^Prioritization ranking: (5) must have, (4) high priority (feasible without), (3) very important (should have), (2) consideration (could have), (1) desirable (will not have at this time).

**Table 3:** Prioritization ranking of the form of the network: user interface, data and security, and other considerations (weighted average scores across patients and families [n=5]; clinicians [n=4]; clinical support service providers [n=5]; and network advisors [n=2]).

Form of the network and its prioritization ranking				Ranking^a^
**User interface**				
	**Must have**			
		Simple or intuitive interface Provide support, guidance, and assistance with how to navigate and use platform (ideal: offer video tutorials) Passes Web Content Accessibility Guidelines (WCAG). Example: large font Ability to easily identify new content (since user’s last login)	5.00	
		Provides IT technical support (for members)	5.00	
		Easy access to support user engagement Smooth and simple login process Optimized for mobile device Real-time information and comments available and accessible (not prescreened by community moderator)	4.53	
	**High importance (feasible without)**			
		Aesthetically refined Pleasing to the eye, organized, and appropriate imagery Symmetrical and aligned (looks modern)	4.00	
		Appropriate use of pop-ups and other interactive elements	4.00	
		Does not allow advertising Does not have advertisements on the platform itself Does not send any unsolicited promotional emails related to the platform or other	3.76	
**Data and security**				
	**Must have**			
		Secure platform or user privacy protected (Health Insurance Portability and Accountability Act [HIPAA] compliant)	5.00	
		Public forum for information and resources, but opportunities for private discussion forums	5.00	
	**Should have (very important)**			
		Data are owned by the co-design team’s institution (not the vendor) Establish terms and conditions for how information or data will be accessed, stored, and used No selling of data to for-profit or not-for-profit entities (pharmaceuticals or other) for financial gains	3.47	
		Data access and analysis Provides actionable metrics related to user activity and engagement (including IP address) Data analysis capabilities available within local database Create an extract of selected data Ability to survey users	3.00	
**Other considerations**				
	**High importance (feasible without)**			
		Health-focused support network Network software is targeted to health-focused communities, has features and functions relevant to health, self-management, etc (eg, health needs assessment) and has experience working in peer-to-peer health care	4.00	
		Sustainability (retaining users) Provides facilitation and network growth support (through designated pump primers, marketing, etc) Opportunity to offer member incentives (through badges, quality improvement initiatives: creating educational material, etc)	4.00	
		Scalability Interactive and responsive; ability to customize and add measures and functionality over time	4.00	

^a^Prioritization ranking: (5) must have, (4) high priority (feasible without), (3) very important (should have), (2) consideration (could have), (1) desirable (will not have at this time).

The most highly prioritized specifications to support each function (Table 2) included providing a supportive and respectful space; providing connections to trusted and curated local, national, and international resources; reducing the difficulty of asking for help by normalizing needing help; and providing curated resources to address the most common concerns (eg, easy access to frequently asked questions and answers). 

The user interface (Table 3) must be simple and intuitive, provide technical support for users, and be easy to access. It was highly important for the user interface to be aesthetically refined, include appropriate use of interactive elements, and not allow external advertising. The platform must be secure and protect user privacy. It must be available as a public forum for information but also allow participants to communicate via discussions not visible to others outside of the network. Other highly important considerations included hosting by a vendor with experience in providing health-focused networks and providing features that support sustainability and scalability (such as member incentives and the ability to customize or add functionality over time).

### Pilot Test Design Specifications

Following the vendor selection process, we worked with CareHubs, an online health network vendor, to build the ConnectShareCare network. The network included (1) a single support community for active and bereaved care partners; (2) a short list of curated resources based on needs identified during the design (planning ahead, practical issues, emotional issues, communication issues, and family resources); (3) a calendar of online and in-person regional support programming; (4) a story from an end user about their experience as a care partner; (5) a roster with member profiles; (6) a help center; and (7) community guidelines. Screenshots are included in Multimedia Appendix 3*.*

The initial feasibility of the network was demonstrated through the active participation of founding members, beginning in April 2021, with expansion to include 12 new care partner members in May 2021 and 15 new members in June 2021. During this period, the network had an average of 135 posts per month. A total of 16 members posted a message to the network, with an average of 11 members posting per month.

## Discussion

### Principal Findings

A co-design process generated a useful and feasible regional, facilitated, and web-based peer support network for care partners of people with serious illnesses. The co-design process ensured that all voices were heard, especially among people who typically may not work together. Design decisions were made collectively and systemically, which allowed network functions and specifications to be identified and prioritized. By doing so, the co-design process ensured that the most critical decisions were responsive to regional needs and preferences. The resulting network connects active and bereaved care partners with peers to facilitate emotional support and exchange information related to caregiving for people with serious illness.

### Comparison With Prior Work

Similar to other networks [15,33,51-62], ConnectShareCare has a clear purpose, includes mechanisms to foster a strong sense of community and support among regional care partners, and provides value to a variety of groups. The network builds on the resources, wisdom, and experiences of care partners. The inclusion of a moderator helps ensure a safe environment that is protected from misinformation, trolling, or cyberbullying. The moderator sets the tone and etiquette with members, modeling behavior and other preventative measures, as well as moderating posts, facilitating connections, and providing feedback to adjust member behaviors [20,23].

Our design process had several strengths. First**,** our process engaged people who would be end users of the network in making critical design decisions [40]. In contrast to asking end users about single decisions, our process allowed end users to make decisions in the context of all other design requirements and options. This led to a more systemic approach to engagement that ensures that decisions are optimized to fit together. Second, our process brought together people with different expertise (in being a person with serious illness or a care partner, in medicine, in health network design, and in community management and moderation) that may typically not work together to create services, and each had different needs to maintain the value of the network. The process also engaged people with engineering design [44,45] and user-centered design [43] expertise to ensure that the process was rigorous enough to produce a network tailored to the needs of end users. Third, our design was responsive to regional needs by addressing gaps in available services and drawing upon local assets. We intentionally worked with multiple health care and support organizations in the region to broaden our network and reach care partners most in need of support, regardless of where formal health care services were received. Care partners are a vulnerable population who often receive minimal structured support from the health system, yet they have significant knowledge to contribute on how to navigate health care systems, health and social resources, and losses at every stage (eg, relationships, identity, and freedom). This knowledge is often actionable by peers. Finally, our process may have supported growth in network participation due to health care and regional support partners feeling heard and included in the design process.

### Limitations

This project has certain limitations that should be considered by those wishing to adapt the methods and findings to different contexts. First, the project was built around a recognition that people who live in north-east rural areas can be particularly isolated and lack access to sources of support. It is unclear whether the network will meet the needs of people in other regions of the country. Second, similar to local demographic characteristics, our design team had limited racial or ethnic diversity. We do not know how greater racial, ethnic, or sociodemographic diversity would enhance or create barriers to its success. Third, the network is built around an asynchronous model. This can be very important because it respects the different schedules that people are on; however, it limits the opportunities for people to hear and see each other in real time. It is unknown whether a network that combines synchronous and asynchronous components would be useful in our context. Finally, while the design process requires extensive back and forth among participants and may not be feasible in other situations, it also represents a strength in creating a network that more closely reflects community needs. In our situation, the decision to create a new network was a result of the recognition that other solutions are not likely to fulfill the needs of our end users.

### Conclusions

Care partners of people with serious illness often lack support and are likely to experience significant challenges and unmet needs. We followed a structured co-design process to collaboratively identify and prioritize the functions and specifications of a regional web-based facilitated peer support network to help care partners cope with the surprises that arrive during serious illness and bereavement. The network was designed to provide emotional support and exchange information related to serious illness caregiving. The coproduction of accessible peer-led information, resources, and support may extend the scope of services offered by a health system to support lay care partners—becoming part of a sustainable, person-centered value-creation system [63,64]. Opportunities exist to evaluate the feasibility of actively engaging community members and moderators in the network [65,66] and will be reported on in a publication under development. Moreover, there is a need to understand effective mechanisms to recruit and retain participants and provide a safe environment to people who are in vulnerable situations; to monitor the network life cycle through metrics related to activity and growth [20,22]; and to consider the creation of network subgroups to support care partners of people with particular illnesses (eg, cancer, dementia, and Parkinson disease) or people from specific minority populations. Finally, there is an opportunity to understand the impact of a regional support network on care partner quality of life, self-confidence, loneliness, and isolation [67,68]; and on health system reputation, use, and visibility.

## Appendix

Multimedia Appendix 1 Surveys administered to active and bereaved caregivers.

Multimedia Appendix 2 Prototype and testing results.

Multimedia Appendix 3 ConnectShareCare screenshots.
