# Supplementary figures and images for: A Web-Based Peer Support Network to Help Care Partners of People With Serious Illness: Co-Design Study
Source: JMIR Hum Factors. 2024 May 8;11:e53194. doi: 10.2196/53194 (PMC11112480; doi:10.2196/53194)

**Appendix 1. Surveys administered to active and bereaved caregivers
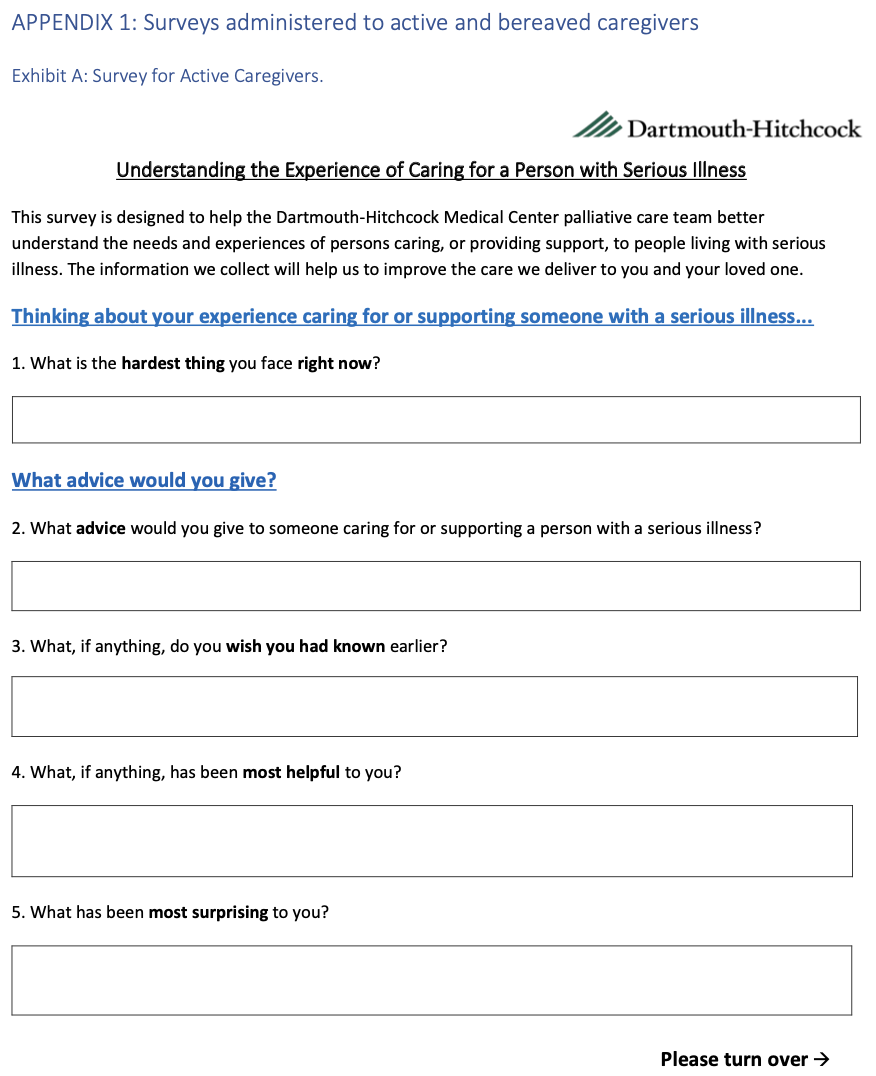

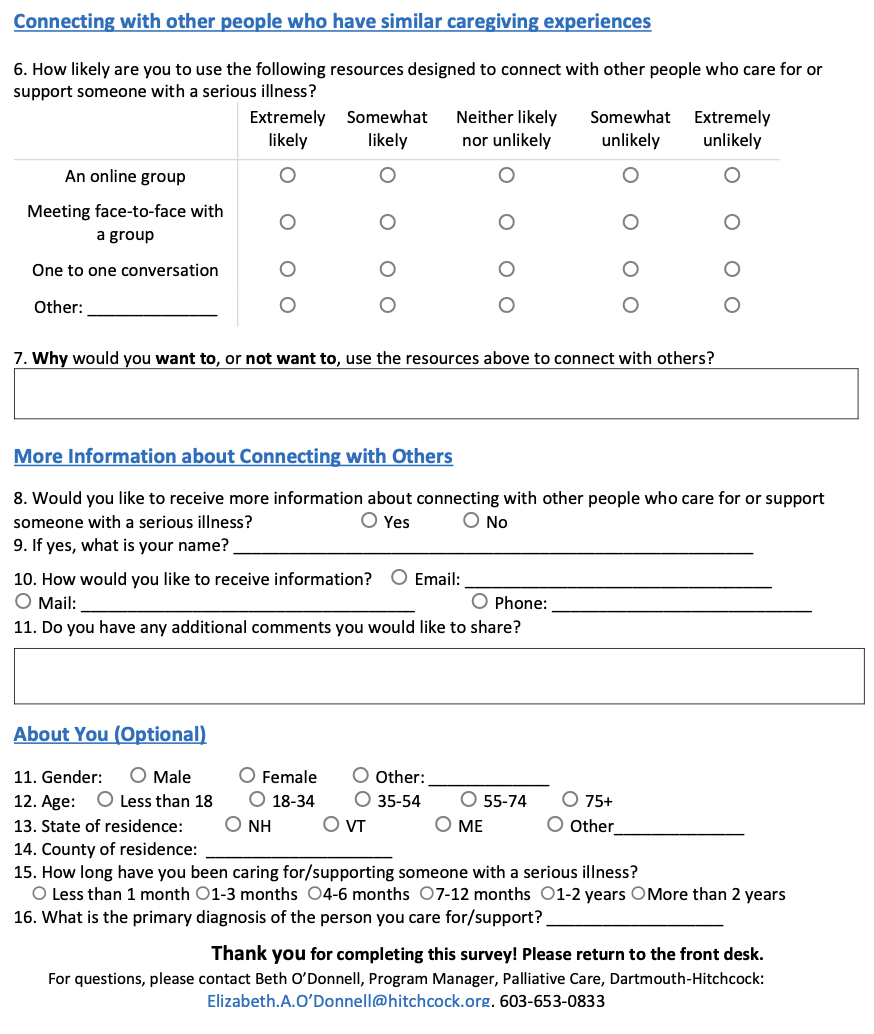
**

**
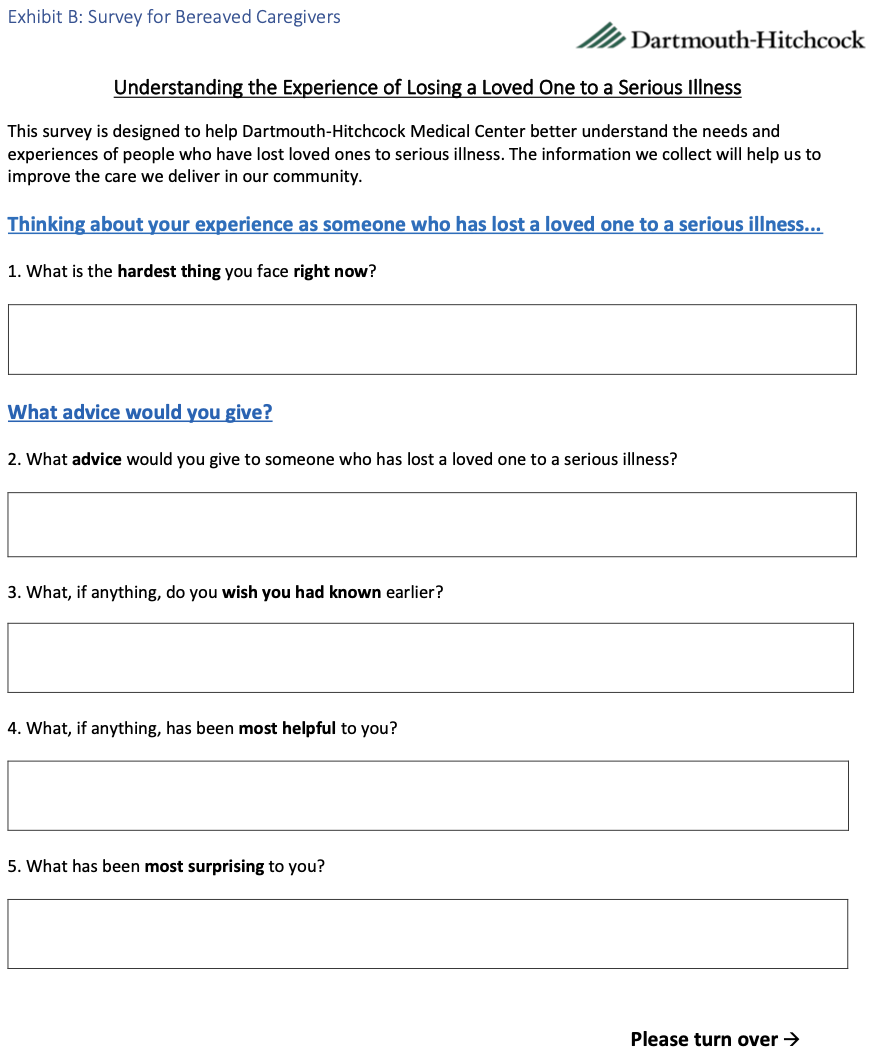
**

**
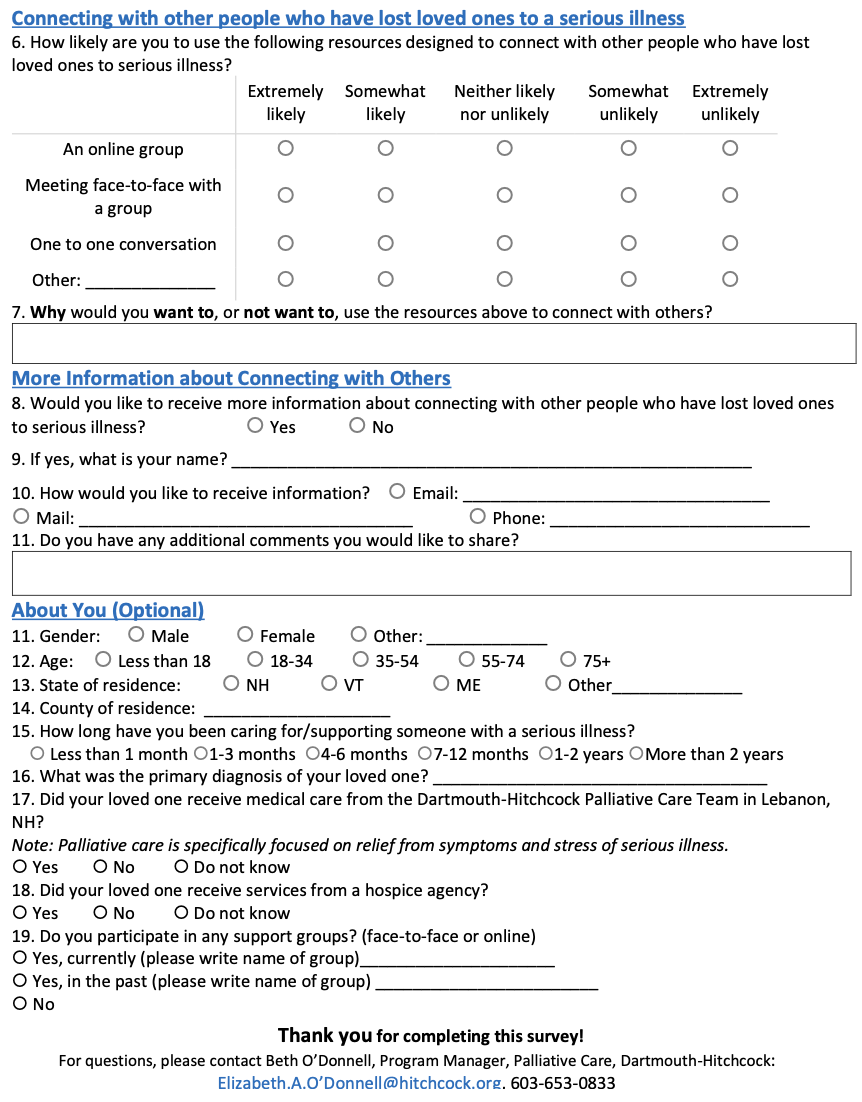
**

Supplement: Multimedia Appendix 1 [file humanfactors_v11i1e53194_app1.docx]
