# Supplementary material for: A Web-Based Peer Support Network to Help Care Partners of People With Serious Illness: Co-Design Study
Source: JMIR Hum Factors. 2024 May 8;11:e53194. doi: 10.2196/53194 (PMC11112480; doi:10.2196/53194)
Supplement: Multimedia Appendix 2 [file humanfactors_v11i1e53194_app2.docx]

**Appendix 2. Prototype and Testing Results**

Below are brief descriptions of what was tried, and what was learned from prototype testing of potential components of the *ConnectShareCare* network.

## Prototype 1: Storytelling

### What we did

Design Team members tested different forms of telling and sharing stories as a mode of connection for those who care(d) for a loved one with serious illness. The different formats included writing stories down and sharing through Google Docs, meeting face-to-face, recording an audio version of the story, recording a video version of the story, and capturing the story through a series of photos. Participants of this prototype were then asked to reflect on the experience of sharing and hearing each other’s stories.

### What we learned

Care partners and families want to share their experiences and be heard. Care partners need to feel that they are in a “safe space” (away from those for whom they care) to tell their story. Enabling videos might support enhanced connection, due to the ability this affords to feel/see emotions on a different level. Different mediums work best for different people, so providing options on how to tell a story seems important. Setting expectations prior is key for a valuable experience, especially for the person telling the story (i.e., do they want advice, just want to vent and/or share, etc.)

*“I was interested in telling [Buddhist friend] how I was feeling about the situation. I chose him because he’s a thoughtful guy. I guess I had hoped that there would be more empathy. It turned out he saw his job was to educate me...it’s important to clarify expectations. Looking back...I hadn’t thought through what I expected--what I was looking for. Now I’m saying I would have looked for empathy.” - Bereaved family care partner, prototype participant*

##

## Prototype 2: Face-to-face group conversations

### What we did

Four care partners (three in bereavement and one actively caregiving) participated in a focus group with two facilitators to test the dynamic of having both active and bereaved care partners in the same group, and to test the dynamics of a mixed-diagnosis group (loved ones have/had different serious illnesses). Another focus group was conducted with only three active care partners.

### What we learned

Overall, these tests were successful: the group dynamic was organic, natural, and genuine. Offering occasional face-to-face interactions to complement an online platform seems critical: in-person is helpful for trust and relationship-building, which might be particularly important to the vulnerable population we aim to support. In any face-to-face event, a skilled moderator is a “must-have.” Care partner needs transcend across disease groups, although certain kinds of questions and issues are rather disease specific. The program should be endorsed by physicians to be optimally successful (not be “just another brochure” which clinicians give out without enthusiasm).

*“I’m really busy, but this kind of event can be really valuable. And no one’s ‘too busy’ for something that’s truly important.” – Active care partner, focus group participant*

##

## Prototype 3: One-to-one matching

### What we did

Design Team members and care partners (both current and bereaved) tested one-to-one "matches", which consisted of an unmonitored one-to-one conversation, either in-person or via telephone. The hypothesis was that this personal and private connection among peers going through similar circumstances will serve to 1. normalize complicated and unexpected emotions, 2. provide emotional support and relief from someone who has really "been there," and 3. facilitate the sharing of knowledge, how-to, and information on available resources.

### What we learned

Overall feedback from matches was very positive. However, expert consultants have cautioned against a one-to-one matching program, citing the high-risk nature of these unmonitored connections and the difficulty of scaling such a program. While a good match is highly valuable to individuals, facilitating a good match is resource intensive. Design team leadership continues to match alike peers to grow network and foster connections.

*“Our conversation was a combination of very practical and very personal experiences. As such, it was extraordinarily valuable for me. I feel much less apprehensive about my ability to honor my husband and support our children when the time comes. I feel very grateful to [the person I spoke with] for being so forthcoming and to you Beth for facilitating our conversation.” – Active care partner*

##

## Prototype 4: Group videoconference

### What we did

We attempted to schedule a facilitated group conversation via video call with 5-8 active care partners, to see whether the benefits of an in-person discussion group can be combined with the convenience of an online solution.

### What we learned

We were unable to host this event due to the low availability of participants, learning that perhaps a videoconference is not as appealing an event as an in-person meeting—unlike our in-person conversations, this one did not seem to meet the threshold of “important enough” for participants to rearrange their busy schedules in order to attend.

##

## Prototype 5: Online forum

### What we did

Design Team members participated in an online discussion forum, Plush Forums, for several months, during which they were asked to post questions and replies to the online message board. The hypothesis was that through asynchronous communication, users would be able to share stories, ask questions, give and receive advice, and share and access resources.

### What we learned

Plush Forum was the design team's first experience with testing an online forum for asynchronous communication. This agile, low-cost prototype allowed for research team members and the Design Team to test out forum functionality, ease of use, and to brainstorm possible facilitators and roadblocks to users engaging with similar online platforms. Overall, we found that forum aesthetics, accessibility, and organization were necessary facilitators to forum use, and that an instruction manual, if possible, in video format, would be useful for forum users. Assuaging security concerns, ensuring ease and simplicity of the sign-up process, and creating a welcoming, safe space for discussion emerged as the key learnings from this test.

*“Once familiar with platform, it’s fun to have discussions with others who are known through our meetings. I don’t use social media, so for me this feels ‘safer’ because I know who’s on it." – Prototype participant*

## Prototype 6: “Caregiver Day” event

### What we did

We held a drop-in, open house event near the 3K waiting area for the Dartmouth Cancer Center. This event focused on appreciation of care partners, relaxation, and building awareness of resources. The hypothesis was that a drop-in event for care partners right outside of the cancer center will be more likely to fit into their day and meet their needs while they are already at the medical center.

### What we learned

We found that, despite reducing access barriers by co-locating near the cancer center, many individuals felt too rushed to stop. Some hurried past us to check in and then spent 20 minutes waiting in 3K, anxious not to miss their appointment. Many expressed appreciation and were touched by the event as a small acknowledgment of care partners and all they do. While the staff and volunteers running the event expressed a spirit of welcoming to all passersby, the actual space could have been more welcoming. Barriers to stop the cold may have been perceived as an effort to keep the event private. In the future, considerations should be made in order to make the space as visible and accessible as possible.
