# Supplementary material for: A Web-Based Peer Support Network to Help Care Partners of People With Serious Illness: Co-Design Study
Source: JMIR Hum Factors. 2024 May 8;11:e53194. doi: 10.2196/53194 (PMC11112480; doi:10.2196/53194)

**Appendix 3. *ConnectShareCare* Screenshots**

*Landing page*


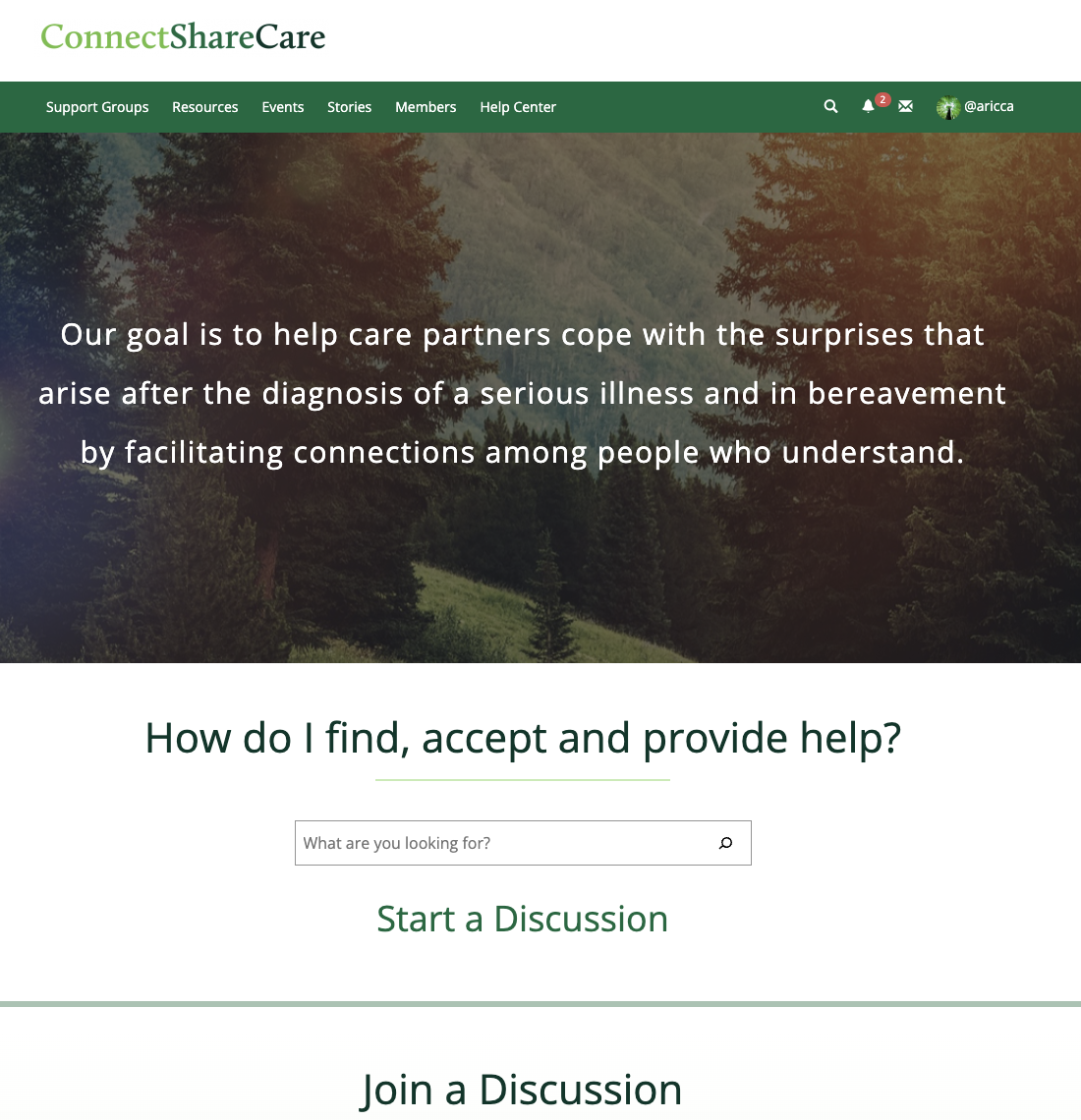


*Resources*


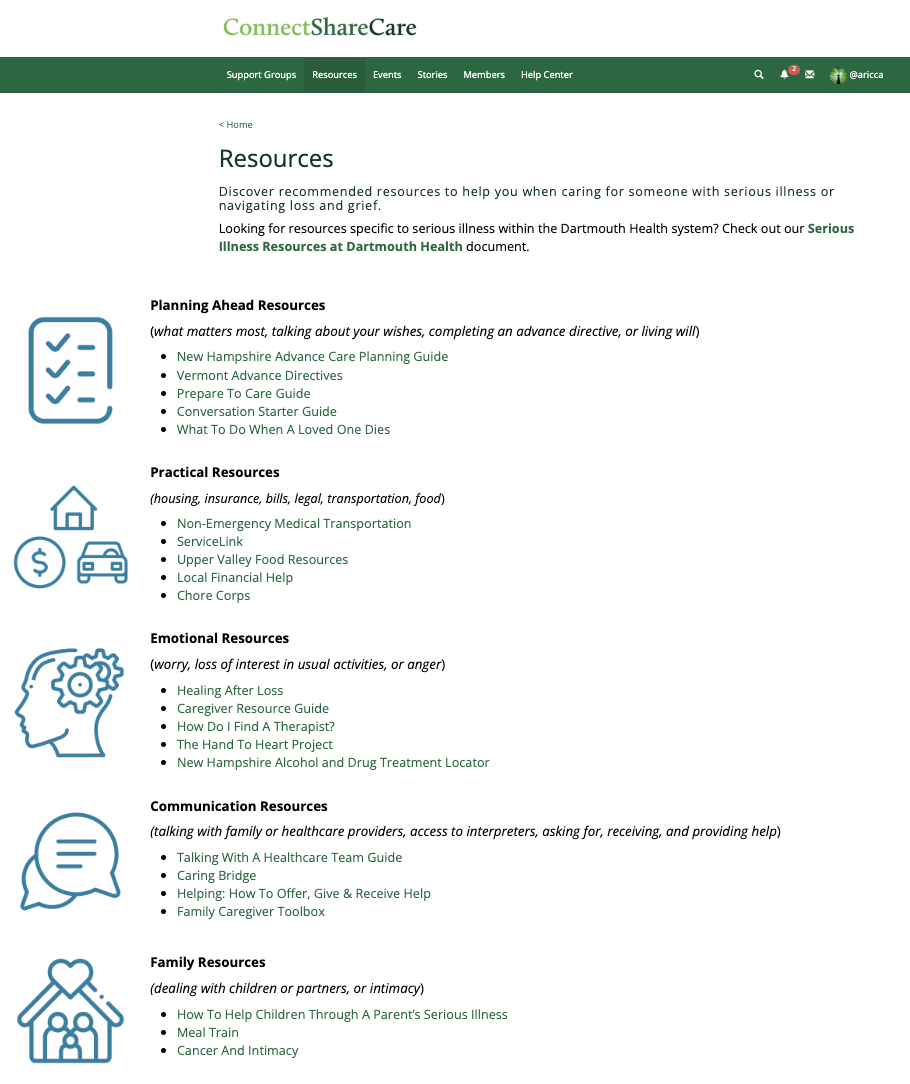


*Events*

**
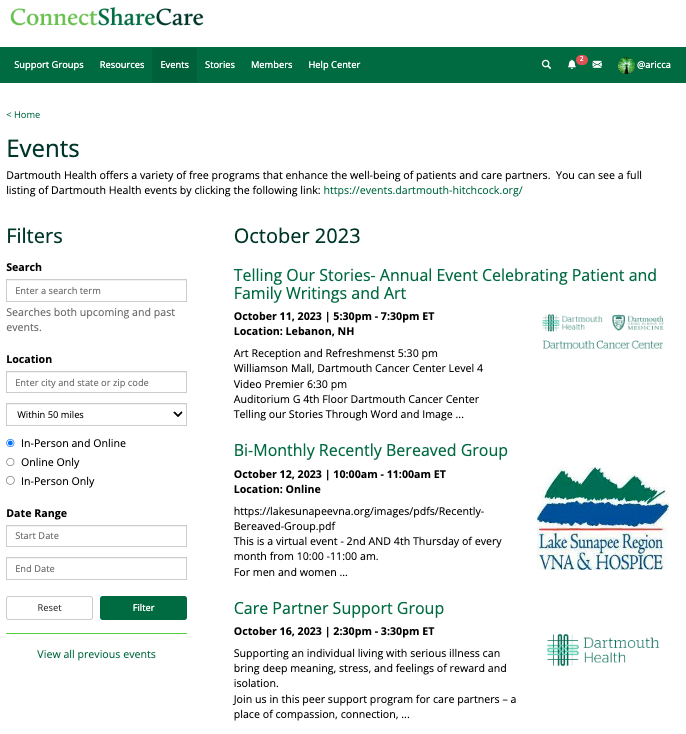
**

*Stories*


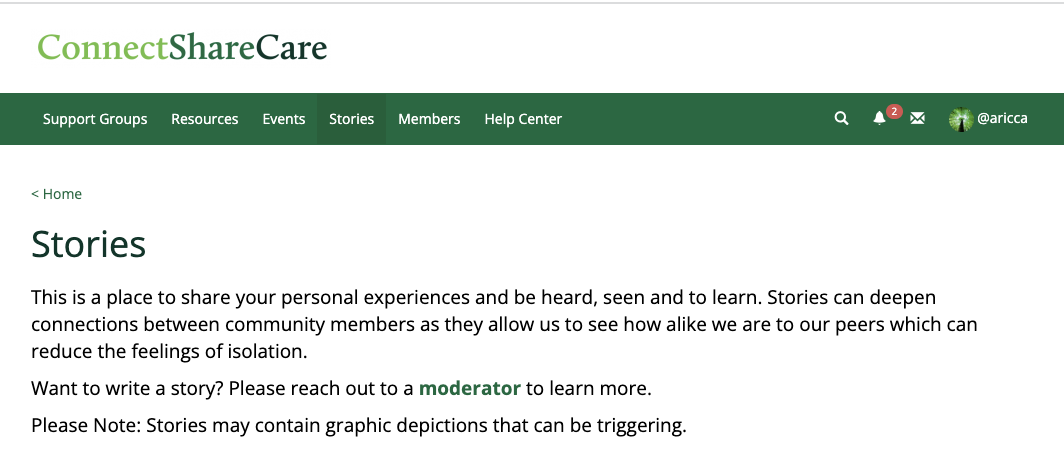


*Help Center*


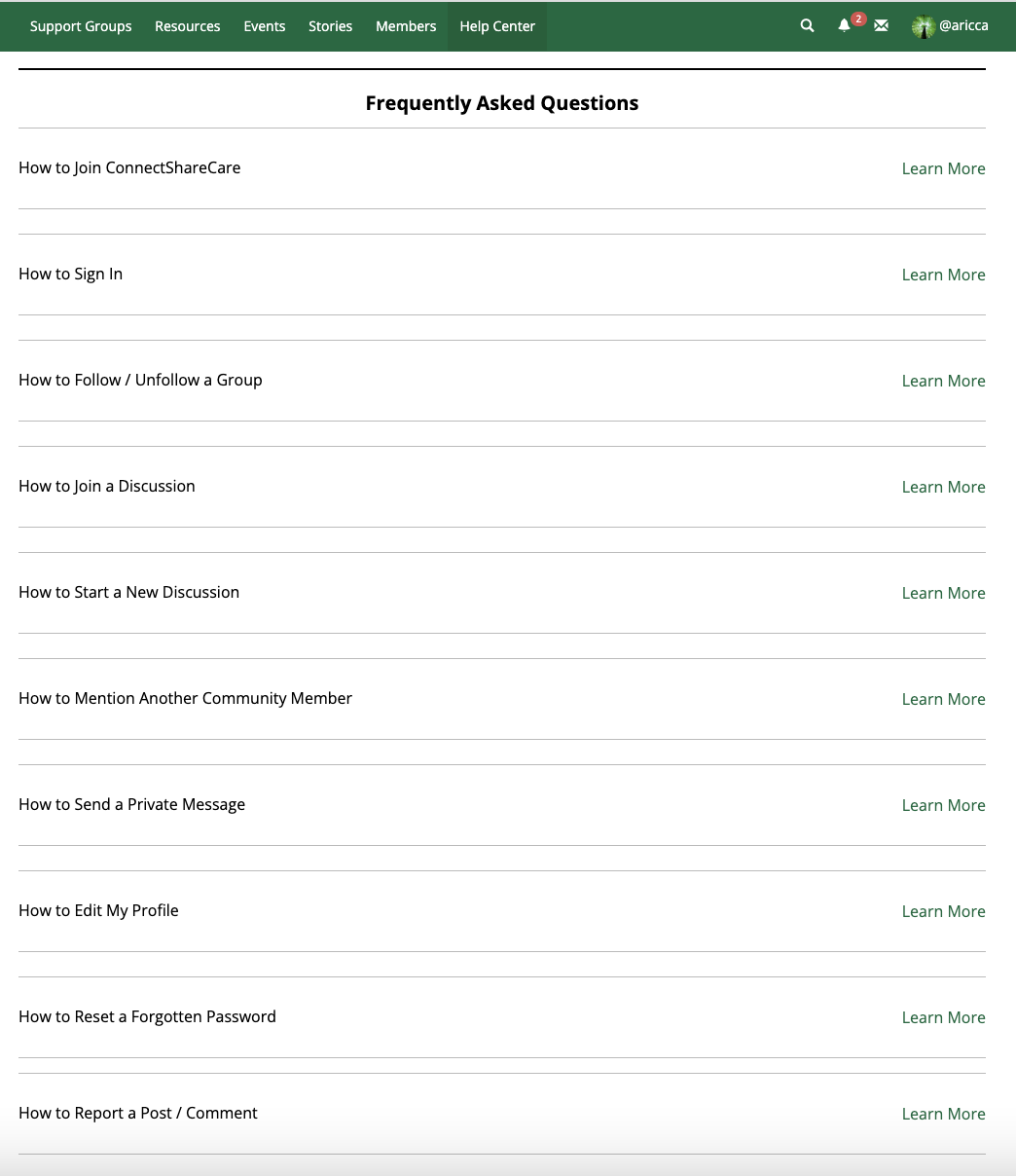


*Community guidelines*


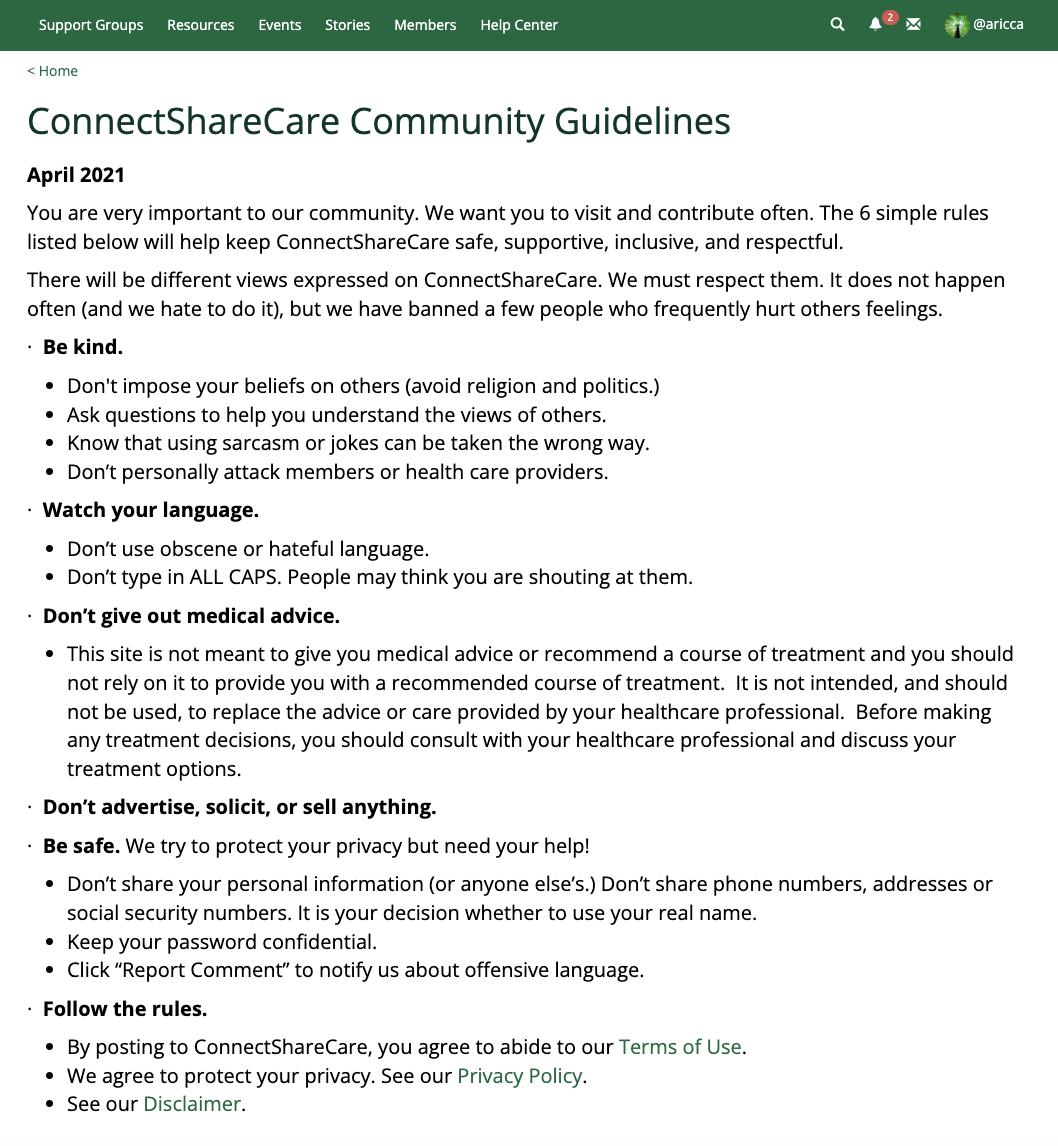

Supplement: Multimedia Appendix 3 [file humanfactors_v11i1e53194_app3.docx]
